# Quality of life-associated factors among patients undergoing coronary artery bypass surgery as measured using the WHOQOL-BREF

**Published:** 2009-10

**Authors:** Mahdi Najafi, Mehrdad Sheikhvatan, MD, Ali Montazeri

**Affiliations:** Department of Anesthesiology, Tehran Heart Centre, Tehran University of Medical Sciences, Iran; Research Department, Tehran Heart Centre, Tehran University of Medical Sciences, Iran; Department of Mental Health, Iranian Institute for Health Sciences Research, ACECR Tehran, Iran

## Abstract

This was a study of the pre-operative factors that influence quality of life (QoL) in patients with coronary artery disease and the relationship between pre-operative QoL and early outcome after coronary artery bypass surgery (CABG). Using the WHOQOL-BREF questionnaire, 283 patients who underwent isolated coronary artery bypass surgery were interviewed and scores were obtained for the physical, psychological, social and environmental components.

The study found that the independent physical component predictors for higher QoL included male gender and diabetes mellitus, while the independent psychological component predictors were male gender and high ejection fraction. Males, diabetics and patients with low education levels had higher social well-being than others. Among the postoperative complications, only respiratory failure was found to have a relationship with physical and psychological components.

Women with coronary artery disease who were candidates for CABG had lower scores than the men in respect of all components of QoL. Furthermore, a lower pre-operative psychological score in patients undergoing CABG can influence postoperative complications, especially respiratory failure.

## Summary

The World Health Organisation defines quality of life (QoL) as ‘an individual’s perception of their position in life in the context of the culture and value systems in which they live and in relation to their goals, expectations, standards and concerns’.^1^ The subjective nature of quality of life means that it can be conceptualised differently by different individuals and groups of people, and is influenced by age, gender, health status and cultural factors, among others.^2^ For the purposes of this study, QoL was defined as a sense of well-being, meaning, and value or self-worth.^1^

It has been demonstrated that the risk factors for coronary artery disease (CAD) influence QoL, and a reduction in exposure to these risk factors would imply lengthening of life and improved health-related QoL in patients. Smoking, closely followed by high alcohol consumption and obesity are most responsible for the loss of QoL in both men and women.^3^ Also, diabetes mellitus, hypertension and dyslipidaemia impact negatively on QoL in patients with cardiovascular disease.^4^,^5^

Patient perception of quality of life is also influenced by therapy.^6^ Treatment of CAD risk factors, such as antihypertension therapy, improves QoL.^7^ Quality of life has also been acknowledged as an important tool in the evaluation of people for entry into drug rehabilitation programmes.^8^,^9^ The evaluation of QoL before coronary interventions can predict postoperative complications and mortality,^10^ while improvement in the components of QoL as well as an overall sense of well-being are related to the improvements in physical status that typically follow cardiac surgery.^11^,^12^

There are, however, few studies that explore the effects of pre-operative risk factors for CAD on different components of QoL in CABG candidates. This study was undertaken to determine the influence of pre-operative QoL factors on patients with CAD and to assess the relationship between pre-operative QoL and early outcome after the operation. The findings are based on the WHOQOL-BREF questionnaire, which was completed by a sample of Iranian patients before CABG.

## Methods

On admission to the surgical ward of the Tehran Heart Centre, patients who were scheduled to undergo isolated CABG between May and September 2006 were invited to participate in the study by the data manager. With their informed consent, 283 patients completed the WHOQOL-BREF questionnaire^13^,^14^ prior to the operation.

The WHOQOL-BREF defines QoL as participants’ perceptions of their position in life in the context of the culture and value systems in which they live and in relation to their goals, expectations, standards and concerns. QoL refers to individual subjective evaluation, which is embedded in a cultural, social and environmental context. This definition of QoL focuses upon respondent perceptions of the effects of disease and health interventions on QoL. The recognition of the multidimensional nature of QoL in the WHOQOL-BREF is based on a four-domain structure: (1) physical health activities of daily living, (2) psychological body image and appearance, (3) social and personal relationships, and (4) environmental–financial resources.

Summation and calculation of the mean score for each domain was done. According to the methodology, we transformed domain scores to a 0- to 100-point scale by using the WHOQOL transformation table.^13^,^14^ According to the mean of these component scores, patients were divided into two groups in each component: mean score = 56 and higher for physical component, mean score = 58 and higher for psychological component, mean score = 59 and higher for social component and, mean score = 56 and higher for environmental component. A higher score on this questionnaire indicates a better quality of life. A study by Nejat *et al.*^15^ demonstrated good to excellent reliability and acceptable validity of this questionnaire in various groups of subjects in Iran.

In addition, each patient’s medical history and early complications after surgery were collected through interviews and by physical examinations. The data analysed included (1) pre-operative variables, namely general characteristics: age, gender, body mass index (BMI) and education level (primary education defined as primary school or less, secondary education characterised as secondary school level, and tertiary education defined as university/college levels or equivalent);^16^ (2) pre-operative risk factors, namely, current smoking history (patient regularly smokes a tobacco product/products one or more times per day or has smoked in the 30 days prior to admission),^17^ alcohol abuse (the use of alcohol despite recurrent adverse consequences),^18^ opium dependence (according to the DSM-IV criteria for substance dependence, daily regular using of substances),^19^ hypercholesterolaemia (total cholesterol = 5.0 mmol/l, HDL cholesterol = 1.0 mmol/l in men, or 1.1 mmol/l in women, triglycerides = 2.0 mmol/l),^20^ family history of CAD (first-degree relatives before the age of 55 in men and 65 years in women),^21^ hypertension (systolic blood pressure = 140 mmHg and/or diastolic = 90 mmHg and/or on antihypertensive treatment),^22^ diabetes mellitus (symptoms of diabetes plus at least one of the following: plasma glucose concentration = 200 mg/dl (11.1 mmol/l), fasting plasma glucose = 126 mg/dl (7.0 mmol/l), plasma glucose two hours postprandial = 200 mg/dl (11.1 mmol/l)),^23^ cerebrovascular disease, and peripheral vascular disease; (3) pre-operative cardiac status, namely, previous myocardial infarction (an acute event with abnormal creatine phosphokinase and troponin levels), Euroscore, and functional class; (4) pre-operative homodynamic status, namely, the number of defective coronary vessels and left ventricular ejection fraction.

In addition, we considered two criteria of a complicated postoperative short-term outcome: (1) in-hospital postoperative complications (existence of at least one of these complications: postoperative arrhythmias, wound infection, stroke and respiratory failure); (2) in-hospital mortality rate (sometimes termed operative mortality), defined as death in hospital before discharge.^24^

After having described the sample and its main characteristics, we explored the variations in QoL subsequent to CABG and the predictors of these variations. Results were reported as the mean ± standard deviation (SD) for quantitative variables and percentages for categorical variables. Groups were compared using the Student’s *t*-test for continuous variables and the chi-squared test (or Fisher’s exact test if required) or Mantel-Haenszel chi-squared test for trend for categorical variables. The predictors exhibiting a statistically significant relationship with the QoL components in the univariate analyses were taken for a multivariate logistic regression analysis to investigate their independence. Odds ratios (OR) and 95% confidence intervals (CI) for OR were calculated.

Model discrimination was measured using the *c* statistics, which is equal to the area under the ROC (receiver operating characteristic) curve. Model calibration was estimated using the Hosmer-Lemeshow (HL) goodness-of-fit statistics (higher *p*-values imply that the model fits the observed data better). The data analyser was anonymous, and data collection and processing were approved by the institutional review board of our heart centre. *P*-values of 0.05 or less were considered statistically significant. All statistical analyses were performed using SPSS version 13 (SPSS Inc, Chicago, IL, USA) and SAS version 9.1 for windows (SAS Institute Inc, Cary, NC, USA).

## Results

Demographic characteristics of patients and common postoperative complications are summarised in Table 1. The most common risk factors for CAD in the patients studied were hyperlipidaemia (68.0%), hypertension (49.1%) and diabetes mellitus (42.2%). The majority of patients had three defective coronary vessels and about half of them were NYHA functional class II. Also, among postoperative complications, the most prevalent complications were arrhythmias (35.4%) and respiratory failure (14.9%).

**Table 1:** Pre-Operative And Postoperative Characteristics Of Patients

*Pre-operative characteristics*	
Male gender	74.2
Mean age (years)	59.8 ± 9.0
Body mass index (kg/m^2^)	27.2 ± 4.3
Education level	
Primary	51.9
Secondary	32.4
Tertiary	15.6
Family history of CAD	46.0
Current cigarette smoking	37.5
Alcohol use	11.6
Opium use	14.9
Hyperlipidaemia	68.0
Hypertension	49.1
Cerebrovascular disease	4.4
Diabetes mellitus	42.2
Peripheral vascular disease	20.4
Last creatinine (mg/dl)	1.3 ± 0.2
Previous myocardial infarction	49.5
Ejection fraction	49.3 ± 9.7
NYHA functional class	
I	33.5
II	51.3
III	15.3
Euroscore	2.3 ± 2.2
Number of defective vessels	
One	3.6
Two	23.3
Three	73.1
Postoperative complications	
Wound infection	1.1
Arrhythmias	35.4
Respiratory failure	14.9
Stroke	0.7
Postoperative mortality	0.7

CAD: coronary artery disease. Data are presented as percentage or mean ± SD

The relationships between QoL component scores and both pre-operative CAD risk factors and postoperative complications were evaluated with univariate analysis (Tables 2, 3, 4, 5). All four physical (*p* < 0.0001), psychological (*p* < 0.0001), social (*p* = 0.023) and environmental (*p* = 0.003) component scores were higher in men than in women. The physical component score in diabetic patients was higher than in non-diabetics (*p* = 0.005). Also, patients with higher ejection fractions had higher psychological scores (*p* = 0.019).

**Table 2:** Comparison Of Patients’ Characteristics Between Physical Component Score ≤ 56 And Higher

*Characteristics*	*Physical score > 56 (n = 124)*	*Physical score ≤ 56 (n = 154)*	p-value*
Male gender	90.3	68.2	< 0.0001
Mean age	59.9 ± 8.6	59.6 ± 9.2	0.810
Body mass index	27.3 ± 4.6	27.1 ± 4.0	0.726
Education level			
Primary	58.6	62.0	0.680
Secondary	26.1	22.5	
Tertiary	15.7	15.1	
Family history of CAD	46.2	44.0	0.716
Current cigarette smoking	35.3	39.7	0.455
Alcohol use	11.8	11.9	0.969
Opium use	16.8	13.9	0.510
Hyperlipidaemia	70.6	64.9	0.322
Hypertension	53.8	45.7	0.187
Cerebrovascular disease	4.2	4.6	0.864
Diabetes mellitus	51.3	34.4	0.005
Peripheral vascular disease	21.8	17.2	0.338
Last creatinine (mg/dl)	1.3 ± 0.2	1.2 ± 0.2	0.655
Previous myocardial infarction	49.6	50.3	0.902
Ejection fraction	49.1 ± 9.8	49.4 ± 9.5	0.823
NYHA functional class			
I	34.5	33.1	0.861
II	50.4	51.7	
III	15.1	15.2	
Euroscore	2.2 ± 2.3	2.4 ± 2.2	0.651
Number of defective vessels			
One	2.5	4.6	0.581
Two	27.7	19.9	
Three	69.6	75.5	
Postoperative complications			
Wound infection	0.8	1.3	0.999
Arrhythmias	38.1	33.1	0.392
Respiratory failure	21.0	9.9	0.011
Stroke	0.8	0.7	0.999
Postoperative mortality	0.8	0.0	0.441

CAD: coronary artery disease. Data are presented as percentage or mean ± SD; *Derived from univariate analysis. Missing rate: 1.76%.

**Table 3:** Comparison Of Patients’ Characteristics Between Psychological Component Score ≤ 58 And Higher

*Characteristics*	*Psychological score > 58 (n = 118)*	*Psychological score ≤ 58 (n = 163)*	p-value*
Male gender	89.8	69.9	< 0.0001
Mean age	59.3 ± 8.6	60.2 ± 9.29	0.442
Body mass index	27.8 ± 4.8	26.7 ± 3.9	0.066
Education level			
Primary	60.4	60.4	0.533
Secondary	27.4	21.4	
Tertiary	12.3	18.2	
Family history of CAD	44.2	46.5	0.708
Current cigarette smoking	35.4	38.8	0.573
Alcohol use	9.7	13.1	0.391
Opium use	13.3	16.3	0.498
Hyperlipidaemia	73.5	63.8	0.091
Hypertension	55.8	44.4	0.064
Cerebrovascular disease	2.7	5.6	0.370
Diabetes mellitus	46.0	39.4	0.274
Peripheral vascular disease	23.9	17.5	0.195
Last creatinine (mg/dl)	1.3 ± 0.2	1.3 ± 0.2	0.398
Previous myocardial infarction	45.1	52.5	0.230
Ejection fraction	50.9 ± 9.0	48.2 ± 10.0	0.019
NYHA functional class			
I	30.1	36.3	0.503
II	54.9	48.1	
III	15.0	15.6	
Euroscore	2.2 ± 2.1	2.5 ± 2.4	0.351
Number of defective vessels			
One	5.3	2.5	0.332
Two	23.9	23.1	
Three	70.8	74.4	
Postoperative complications			
Wound infection	0.9	1.3	0.999
Arrhythmias	32.7	37.1	0.458
Respiratory failure	8.8	18.8	0.023
Stroke	0.0	1.3	0.513
Postoperative mortality	0.0	0.6	0.270

CAD: coronary artery disease. Data are presented as percentage or mean ± SD. *Derived from univariate analysis. Missing rate: 0.7%.

**Table 4:** Comparison Of Patients’ Characteristics Between Social Component Score ≤ 59 And Higher

*Characteristics*	*Social score > 59 (n = 118)*	*Social score ≤ 59 (n = 143)*	p-value*
Male gender	87.3	76.2	0.023
Mean age	60.4 ± 8.7	59.5 ± 9.2	0.401
Body mass index	27.5 ± 4.4	26.9 ± 4.2	0.254
Education level			
Primary	67.9	52.7	0.010
Secondary	21.1	26.0	
Tertiary	11.0	21.4	
Family history of CAD	47.9	44.4	0.587
Current cigarette smoking	35.0	38.2	0.599
Alcohol use	9.4	12.5	0.434
Opium use	11.1	15.4	0.314
Hyperlipidaemia	69.2	66.2	0.605
Hypertension	53.0	44.9	0.197
Cerebrovascular disease	3.4	5.1	0.502
Diabetes mellitus	47.9	35.5	0.096
Peripheral vascular disease	21.4	19.9	0.766
Last creatinine (mg/dl)	1.2 ± 0.2	1.3 ± 0.2	0.202
Previous myocardial infarction	45.3	55.9	0.093
Ejection fraction	49.7 ± 9.1	49.0 ± 10.0	0.579
NYHA functional class			
I	35.9	31.6	0.298
II	50.4	50.0	
III	13.7	18.4	
Euroscore	2.4 ± 2.3	2.3 ± 2.2	0.778
Number of defective vessels			
One	3.4	3.7	0.649
Two	25.6	22.1	
Three	70.9	74.3	
Postoperative complications			
Wound infection	0.9	1.5	0.999
Arrhythmias	33.3	37.0	0.540
Respiratory failure	12.0	17.6	0.207
Stroke	0.0	1.5	0.501
Postoperative mortality	0.0	0.7	0.310

CAD: coronary artery disease. Data are presented as percentage or mean ± SD. *Derived from univariate analysis. Missing rate: 7.77%

**Table 5:** Comparison Of Patients’ Characteristics Between Environmental Component Score ≤ 56 And Higher

*Characteristics*	*Environmental score > 56 (n = 109)*	*Environmental score ≤ 56 (n = 169)*	p-value*
Male gender	87.2	72.2	0.003
Mean age	59.8 ± 9.0	59.9 ± 8.9	0.949
Body mass index	27.5 ± 4.4	27.0 ± 4.3	0.333
Education level			
Primary	61.2	61.0	0.864
Secondary	22.4	24.5	
Tertiary	16.3	14.5	
Family history of CAD	47.2	44.2	0.629
Current cigarette smoking	34.0	39.6	0.347
Alcohol use	10.4	11.6	0.758
Opium use	12.3	14.6	0.580
Hyperlipidaemia	67.9	68.3	0.949
Hypertension	53.8	46.3	0.233
Cerebrovascular disease	5.7	3.7	0.548
Diabetes mellitus	42.5	42.1	0.951
Peripheral vascular disease	18.9	21.3	0.622
Last creatinine (mg/dl)	1.2 ± 0.2	1.3 ± 0.2	0.229
Previous myocardial infarction	43.4	53.7	0.100
Ejection fraction	50.5 ± 9.4	48.6 ± 9.9	0.114
NYHA functional class			
I	34.9	32.9	0.322
II	53.8	49.4	
III	11.3	17.7	
Euroscore	2.1 ± 2.2	2.5 ± 2.2	0.206
Number of defective vessels			
One	2.8	4.3	0.964
Two	24.5	22.0	
Three	72.6	73.8	
Postoperative complications			
Wound infection	0.9	1.2	0.999
Arrhythmias	34.0	36.2	0.708
Respiratory failure	14.2	15.2	0.805
Stroke	0.0	1.2	0.521
Postoperative mortality	0.0	0.6	0.246

CAD: coronary artery disease. Data are presented as percentage or mean ± SD. *Derived from univariate analysis. Missing rate: 1.76%

Patients with higher body mass index (*p* = 0.066) tended to have a high psychological score. Patients with lower levels of education had a greater sense of well-being from their social relationships and reported higher levels of sociability (*p* = 0.010).

Multivariate logistic regression analysis showed that among the pre-operative characteristics of patients, male gender (*p* < 0.0001) and diabetes mellitus (*p* = 0.002) for the physical component and male gender (*p* = 0.0001) and high ejection fraction (*p* = 0.018) for the psychological component were independent predictors of higher QoL. Also, men (*p* = 0.032), diabetics (*p* = 0.037) and patients with low education levels (*p* = 0.025) had a higher sense of social well-being than other patients (Table 6). Among postoperative complications, a relationship was found only between respiratory failure and the physical (*p* = 0.011) and psychological (*p* = 0.023) components, while no relationships were found between other early complications and the four components of QoL.

**Table 6:** Factors Influencing Physical, Mental And Social Components Scores Of QOL

*Component scores*	*Variables*	*OR (95% confidence interval)*	p*-value*
Physical component score	Female	1.0 (reference)	
	Male	4.611 (2.278–9.333)	< 0.0001
	No diabetes mellitus	1.0 (reference)	
	Diabetes mellitus	2.264 (1.348–3.802)	0.0020
Psychological component score	Female	1.0 (reference)	
	Male	3.688 (1.843–7.378)	0.0001
	Ejection fraction	1.032 (1.005–1.059)	0.0189
Social component score	Female	1.0 (reference)	
	Male	2.383 (1.169–4.857)	0.0325
	No diabetes mellitus	1.0 (reference)	
	Diabetes mellitus	1.768 (1.032–3.028)	0.0370
	Primary education level	1.0 (reference)	
	Secondary level	0.614 (0.325–1.160)	
	Tertiary level	0.371 (0.172–0.798)	0.0257

For physical component: Hosmer-Lemeshow statistic, χ^2^ = 0.1165, *p* = 0.9434, area under the ROC curve *c* = 0.66915. For psychological component: Hosmer-Lemeshow statistic, χ^2^ = 5.0536, *p* = 0.5370, area under the ROC curve *c* = 0.64876. For social component: Hosmer-Lemeshow statistic, χ^2^ = 4.7882, *p* = 0.4423, area under the ROC curve *c* = 0.63695.

## Discussion

Modern treatments such as CABG in patients with CAD focus not only on improving life expectancy, symptoms and functional status, but also on several aspects of QoL. An improvement in QoL is considered to be important as a primary outcome and in the determination of therapeutic benefit.^25^,^26^ While the WHOQOL-BREF is one of the main tools that can be used to evaluate QoL, in Iran, patients undergoing CABG are not evaluated on this questionnaire. Therefore, in the present study, we considered the role of the WHOQOL-BREF questionnaire in the evaluation of different QoL components before cardiac surgery and their relationships with pre-operative risk factors.

In this study, we found that the men’s scores were significantly higher than the women’s in all four components of QoL. According to Dias *et al*.,^27^ men had a better perception of QoL than women, while women more frequently had a below-median physical score. In their study, female gender was a main independent predictor of both physical and psychological components of QoL in patients with acute coronary syndrome.^27^ In another study, it was indicated that women had more physical, social and psychological dysfunction, and poorer overall health than men.^28^ Rankin *et al*. found that women had more in-hospital complications, cardiac dysfunction and mortality than men.^29^ Other studies have reported that men had improved outcomes over women.^30^,^31^ However, more recent studies have reported that long-term outcomes for men and women were similar.^32^

It has also been demonstrated that a multi-component lifestyle-changing programme focusing on diet, exercise, stress management and social support can successfully improve several components of QoL in women who underwent coronary interventions. 28 In fact, it seems that the gender differences in QOL can be explained by the position of women in relation to men in the family. Here factors such as women’s sense of inability to care for others when they are ill or the extent and nature of support and care available to them when they, as care givers, become ill may be at play.

In our study, a high physical score was found in diabetic patients. In a study by Botija Yagüe *et al*.,^33^ diabetes impacted negatively on all dimensions of their quality of life and the diabetic patients were not positively affected by intensive therapy for cardiovascular risk factors.^33^ Pischke^34^ and Dias^27^ reported that in diabetic patients, especially women, poorer physical functioning was observed when compared to non-diabetics. In Herlitz’s study, physical and psychological scores were similar in diabetic and non-diabetic patients.^35^ However, higher physical and psychological scores in diabetics versus non-diabetics are unusual findings and deserve more investigation. Besides, the assessment of the efficacy of the WHOQOL-BREF questionnaire is necessary in more studies on diabetic patients.

Chan *et al*.^36^ suggested that level of education has no significant bearing on the quality of life of patients with coronary artery disease. By contrast, we found that patients with lower education levels had higher social component QoL scores. This difference may be accounted for, in part, by the impact of education (and class) on patient expectations. It seems that the patients with higher levels of education were less satisfied with themselves and their personal relationships as well as other domains of social functioning.^37^ By contrast, the patients with lower levels of education were more satisfied with their lives and had a greater sense of well-being. These findings on the relationship between education, QoL and health outcomes would have to be tested further in population-based studies.

Another important finding of this study is that there was a strong relationship between both the physical and psychological QoL components and postoperative respiratory failure. Patients with respiratory failure had lower pre-operative psychological scores. There are many studies on the effect of respiratory failure on QoL. However, we found no study on the effects of low scores of QoL on respiratory function. In a study by Euteneuer *et al*, it was indicated that in patients with chronic respiratory failure after long-term mechanical ventilation, physical health was markedly reduced compared to the general population norm, but mental health was only mildly impaired.^38^ Patients with respiratory failure are seriously handicapped mentally and physically compared with those with other diseases. This is because their disorders relate to breathing, which is the basic physical function directly associated with sustaining life.^39^

In general, we found that women with coronary disease who became candidates for CABG had lower scores than men in all QoL components. Education levels influenced patient QoL albeit not in an obvious way. Also, diabetic patients may have had better physical functioning than non-diabetics. This latter finding requires further investigation, as it is unexpected. Lastly, lower pre-operative psychological scores in patients undergoing CABG can influence postoperative complications, especially respiratory failure.
